# Disseminated and Congenital Toxoplasmosis in a Mother and Child With Activated PI3-Kinase δ Syndrome Type 2 (APDS2): Case Report and a Literature Review of Toxoplasma Infections in Primary Immunodeficiencies

**DOI:** 10.3389/fimmu.2019.00077

**Published:** 2019-02-14

**Authors:** Djuro Karanovic, Ian C. Michelow, Anthony R. Hayward, Suk See DeRavin, Ottavia M. Delmonte, Michael E. Grigg, Adam Kerry Dobbs, Julie E. Niemela, Jennifer Stoddard, Zaid Alhinai, Natasha Rybak, Nancy Hernandez, Stefania Pittaluga, Sergio D. Rosenzweig, Gulbu Uzel, Luigi D. Notarangelo

**Affiliations:** ^1^Laboratory of Clinical Immunology and Microbiology, National Institute of Allergy and Infectious Diseases (NIAID), National Institutes of Health (NIH), Bethesda, MD, United States; ^2^Division of Infectious Diseases, Department of Pediatrics, Brown University and Rhode Island Hospital, Providence, RI, United States; ^3^Division of Allergy and Immunology, Department of Pediatrics, Brown University and Rhode Island Hospital, Providence, RI, United States; ^4^Laboratory of Parasitic Diseases, National Institute of Allergy and Infectious Diseases (NIAID), National Institutes of Health (NIH), Bethesda, MD, United States; ^5^Immunology Service, Department of Laboratory Medicine, NIH Clinical Center, Bethesda, MD, United States; ^6^Division of Infectious Diseases, Department of Medicine, Brown University and The Miriam Hospital, Providence, RI, United States; ^7^Department of Medicine and Pediatrics, Brown University and Rhode Island Hospital, Providence, RI, United States; ^8^Center for Cancer Research, National Cancer Institute (NCI), National Institutes of Health (NIH), Bethesda, MD, United States

**Keywords:** immunodeficiency, toxoplasmosis, APDS2, PI3K3R1, PI3K

## Abstract

Phosphoinositide 3-kinase (PI3K) plays an integral role in lymphocyte function. Mutations in *PIK3CD* and *PIK3R1*, encoding the PI3K p110δ and p85α subunits, respectively, cause increased PI3K activity and result in immunodeficiency with immune dysregulation. We describe here the first cases of disseminated and congenital toxoplasmosis in a mother and child who share a pathogenic mutation in *PIK3R1* and review the mechanisms underlying susceptibility to severe *Toxoplasma gondii* infection in activated PI3Kδ syndrome (APDS) and in other forms of primary immunodeficiency.

## Introduction

Phosphoinositide 3-kinases (PI3Ks) control essential functions in cellular activation, development and differentiation through generation of phosphatidylinositol (3,4,5)-trisphosphate (PIP_3_) (1). In lymphocytes, the PI3K complex is made up of a heterodimer consisting of a catalytic subunit (p110δ) and a regulatory subunit (p85α) that are encoded by the *PIK3CD* and *PIK3R1* genes, respectively (1). Heterozygous gain-of-function mutations in *PIK3CD* lead to constitutive activation of the PI3K pathway and cause activated PI3K delta syndrome type 1 (APDS1); similarly, heterozygous *PIK3R1* mutations that affect interaction of p85α with p110δ also lead to constitutive activation of PI3K and cause APDS2 (2–7). The clinical presentation of these primary immunodeficiencies (PIDs) may include recurrent upper respiratory tract infections often leading to bronchiectasis, humoral immunodeficiency with elevated IgM, diffuse lymphadenopathy, Epstein Barr virus, and/or cytomegalovirus viremia, and an increased risk of lymphoma (8, 9). In addition, mutations in the donor splice site in intron 11 of *PIK3R1*^Δ*434* _*475*^, resulting in exclusion of exon 11 from the cDNA, cause APDS2, *s*hort stature-*h*yperextensibility of joints-*o*cular depression-*R*ieger anomaly-*t*eething delay (SHORT) syndrome, or a combination of the two (10).

We describe a female with APDS2 manifesting with short stature, diffuse lymphadenopathy, recurrent upper respiratory tract infections, elevated IgM and disseminated toxoplasmosis who gave birth to a genetically affected daughter with severe, congenital toxoplasmosis.

### Case Report

**Figure 1:**
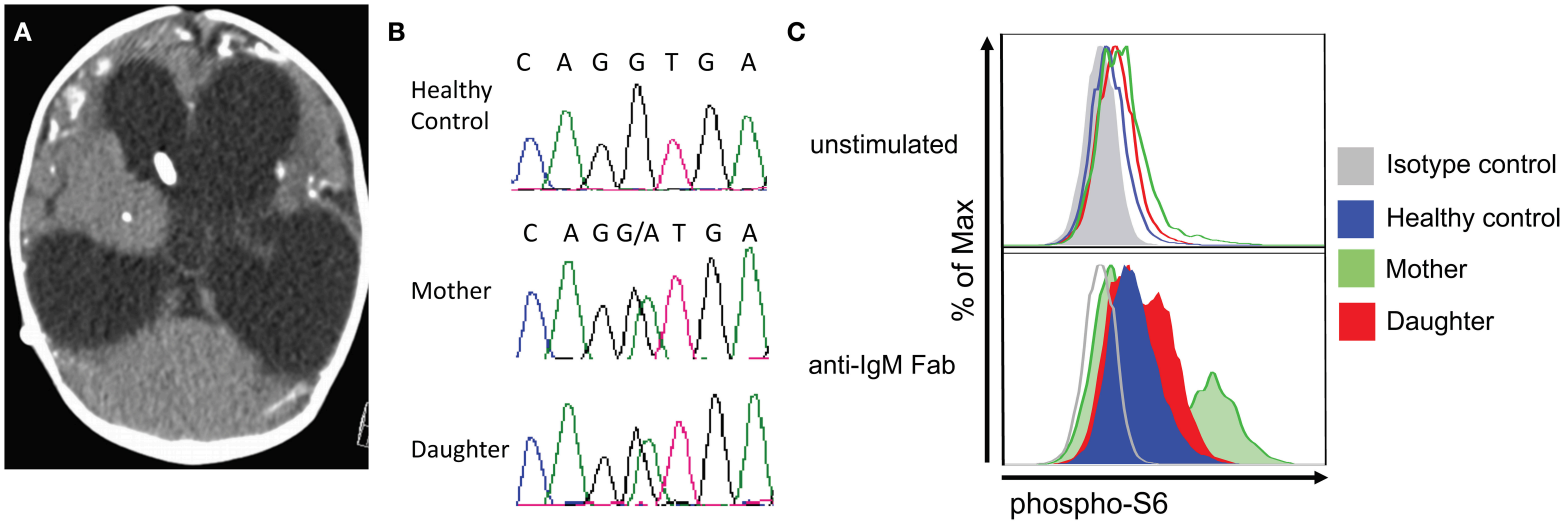
Clinical and laboratory features in mother and daughter with Toxoplasmosis and APDS2. **(A)** Brain CT in the patient's daughter at 3 months of age, showing marked hydrocephalus with enlarged lateral and third ventricles, profound brain atrophy and basal ganglia calcifications. **(B)** Chromatogram demonstrating heterozygosity for the c.1425+1g > a at the *PIK3R1* locus in the patient and her daughter. **(C)** Analysis of phospho-S6 in CD20^+^ cells from a healthy control, the mother, and the daughter at resting conditions (top) and upon *in vitro* activation with anti-IgM (bottom).

The patient was born to non-consanguineous healthy parents of Hispanic and East Asian descent. In early childhood, she was diagnosed with recurrent sinopulmonary and otitis infections. Due to persistent diffuse lymphadenopathy since age 5, she underwent multiple lymph node and bone marrow biopsies that were negative for malignancy. She also suffered from multiple episodes of Herpes Simplex oral infection and genital warts, which required electrosurgical excision of precancerous lesions. At the age of 40, she complained of painful cervical lymphadenopathy and was diagnosed with toxoplasmosis based on positive IgM serology results. An ophthalmological and MRI exam were normal. She was treated for 2 weeks with an unknown agent(s) with the presumption that her infection was cured. Then, she immigrated to Rhode Island (USA), where she gave birth to a female baby at 37 weeks gestation. There were concerns for craniosyntosis, occasional episodes of staring and myoclonic jerks that led to a brain CT scan at 3 months of age that showed marked hydrocephalus, enlarged lateral and third ventricles, and extensive cerebral atrophy (Figure 1A). There were widespread calcifications at the gray-white matter interface and in the basal ganglia. The clinical, laboratory and radiologic features were consistent with a congenitally acquired infection. Tests for CMV, HIV, HSV and syphilis were negative while serologic tests for *Toxoplasma gondii* were positive (IgG was 1:512, IgM was positive by the ISAGA method, IgA antibodies were negative; Palo Alto Toxoplasma Serology Laboratory). *T. gondii* PCR assays of the blood and CSF were negative. A dried blood spot from the newborn screen was positive when retrospectively tested for *T. gondii* IgM antibodies at Massachusetts Department of Health.

The child met criteria for congenital toxoplasmosis (11) and was treated with oral pyrimethamine, sulfadiazine and leucovorin. During the following year, the child had refractory seizures despite treatment with topiramate, levetiracetam and clonazepam, her microcephaly progressed to <1st percentile, and static encephalopathy with poor feeding necessitated a gastrostomy tube. The anti-toxoplasma IgG titer decreased while on antimicrobial therapy and was undetectable by 36 weeks of treatment. Two months after completion of a 1-year course of anti-parasitic therapy, repeat anti-*Toxoplasma* IgG testing showed a rebound to a titer of 1:8,000. At 2 years of age, repeat anti-*Toxoplasma* IgG (1:3,072) and IgM (7.6, normal < 2.0) levels remained elevated. She has elevated serum IgG (1,399 mg/dL) and IgM (215 mg/dL) and undetectable IgA. Her length has consistently remained below the 3rd percentile.

When the child was hospitalized at age 4 months, the mother was not acutely ill, but she had chronic non-tender bilateral cervical lymphadenopathy. Her laboratory tests were significant for strongly positive toxoplasmosis serology thought to be secondary to ongoing chronic infection (IgG was 1:16,000; IgG avidity was high, IgM ELISA was 4.1 (normal < 2.0), and AC/HS ratio of 1,600/3,200). A cervical lymph node biopsy was positive for toxoplasma PCR and she was started on oral pyrimethamine, sulfadiazine, and leucovorin. After 7 months of treatment and moderate improvement in lymphadenopathy, she was switched to suppressive therapy with trimethoprim-sulfamethoxazole (TMP/SMX). When this suppressive regimen was discontinued, the lymphadenopathy worsened. To evaluate for a potential underlying immunodeficiency, both the mother and her daughter were enrolled in NIH protocol 05-I-0213 upon informed consent.

At age 42, the mother was noted to be short (148 cm, <3rd percentile), and to have generalized lymphadenopathy. A mild persistent EBV viremia (up to 2.58log10) and an intermittent CMV viremia (< 3.08log10) was observed.

Immunological investigations revealed normal IgG (986 mg/dL) and IgA (69 mg/dL), with elevated IgM (571 mg/dL). The total lymphocyte count was 1,950 cells/μL. Analysis of lymphocyte subsets by flow cytometry demonstrated decreased CD20^+^ CD27^+^ memory B cells (6 cells/μL), increased proportion of CD19^+^ CD10^+^ transitional B cells (36.4% of total B cells), and lack of CD20^+^ CD27^+^ IgM^−^ switched memory B cells. Specific antibody responses to *Streptococcus pneumoniae* were not protective to all serotypes. T-cell studies were significant for markedly reduced number of naïve CD4^+^ CD62L^+^ CD45RA^+^ cells (10 cells/μL) and increased number of central (CD62L^+^ CD45RA^−^, 265 cells/μL) and effector memory (CD62L^−^ CD45RA^−^, 456 cells/μL) CD8^+^ cells.

Whole exome gene sequencing with targeted analysis of 362 PID genes (Table 1) identified a heterozygous mutation at an essential donor splice site of *PIK3R1* (NM_181523.2:c.1425+1g> a), which was confirmed with Sanger sequencing (Figure 1B). The mutation results in the skipping of exon 11, which encodes a part of the inter-SH2 domain of the regulatory p85α subunit, and results in hyperactivation of the PI3K pathway (6). DNA analysis of the patient's daughter demonstrated the same *PIK3R1* c.1425+1g>a mutation.

**Table 1:** Rare genomic variants identified by whole exome sequencing (WES) and targeted analysis of Primary Immune Deficiency genes in the mother with disseminated Toxoplasmosis.

**Chromosome**	**Gene**	**Variant**	**Zygosity**	**Frequency in ExAC**
1	*FLG*	NM_002016 c.2365C >A p.R789S	het	0
1	*IL12RB2*	NM_001258215 c.778C >T p.R260W	het	8.24 e-6
2	*TTC7A*	NM_001288953 c.415+3A >G	het	0.0007
3	*TFRC*	NM_001313965 c.67A >G p.T23A	het	0.0007
4	*TLR10*	NM_001017388 c.2314C >T p.R772X	het	0.0016
5	*SPINK5*	NM_001127698 c.1764T >G p.I588M	het	0.0028
5	*ERBB2IP*	NM_001006600 c.3260A >G p.N1087S	het	0.0008
5	*PIK3R1*	NM_181523 c.1425+1G >A	het	0
6	*TRAF3IP2*	NM_001164281 c.649C >A p.P217T	het	0.0066
6	*TNFAIP3*	NM_001270507 c.374C >T p.A125V	hom	0.0017
6	*PGM3*	NM_001199918 c.883A >C p.T295P	het	0
8	*VPS13B*	NM_017890 c.2825-4T >A	het	0.0001
16	*NOD2*	NM_001293557 c.2042G >A p.R681H	het	0.002
16	*PLCG2*	NM_002661 c.1712A >G p.N571S	het	0.0066
17	*STAT3*	NM_003150 c.1381G >C p.V461L	het	0.0042
19	*TYK2*	NM_003331 c.590G >A p.R197H	het	0.0027

*Synonymous variants and those with a minor allele frequency (MAF) in ExAC > 0.01 were filtered out. het, heterozygous; hom, homozygous*.

The phosphorylation of Akt is an important downstream event in the activation of the PIK3 pathway. In turn, phosphorylated Akt activates mTOR which phosphorylates S6. Functional studies on the patient's CD8^+^ and her daughter's CD20^+^ cells confirmed an increased S6 phosphorylation after IL-2 and anti-IgM stimulation (Figure 1C), which suggests hyperactivation of the PI3K-mTOR signaling pathway.

**Figure 2:**
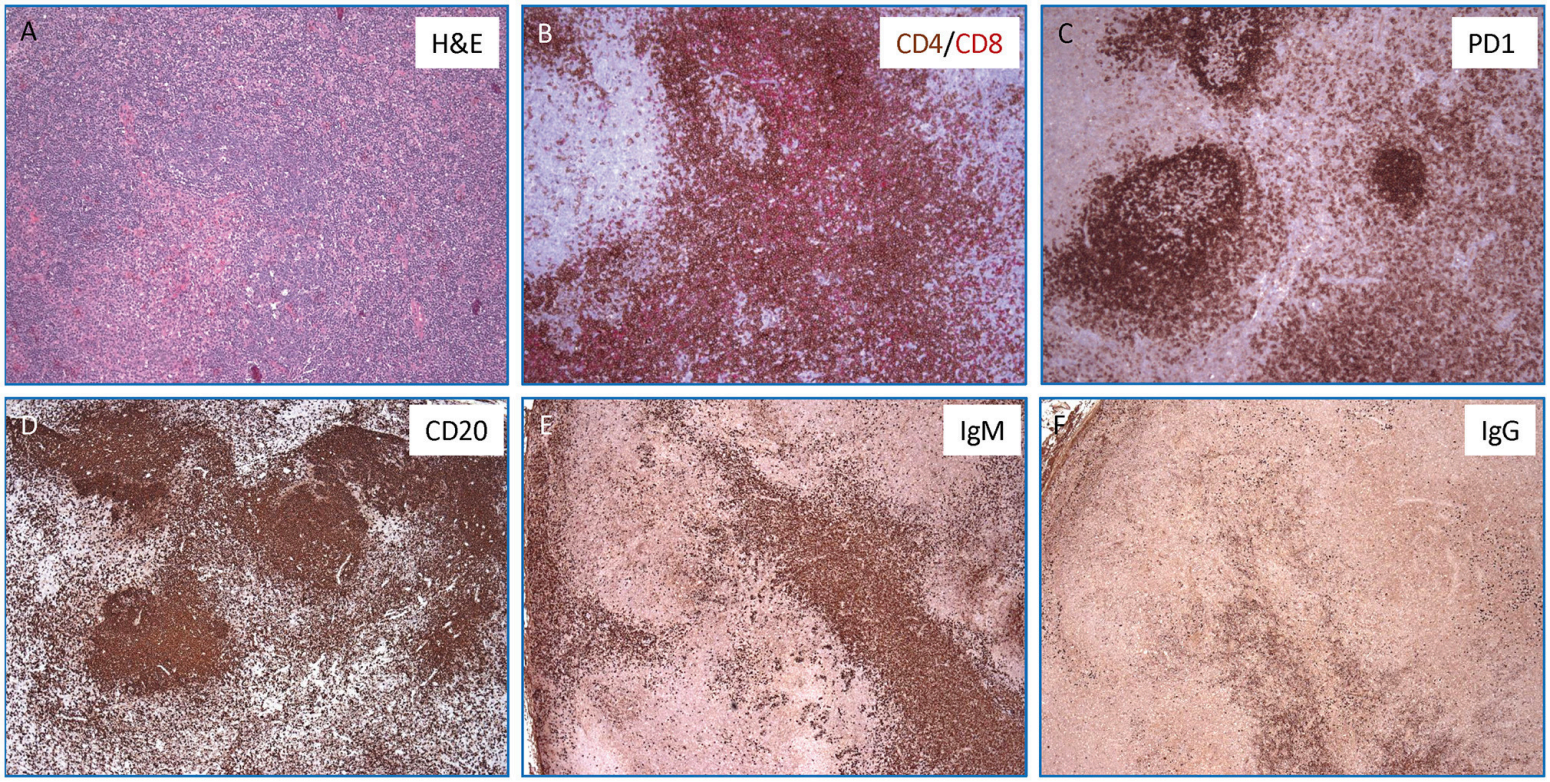
Lymph node histopathology in the mother with APDS2. **(A)** Hematoxylin & Eosin stained section show an ill-defined secondary follicle with “naked”germinal center; bottom left shows a cluster of monocytoid cells with pale cytoplasm (magnification, 10x). In the inlet, CMV positive cells are identified by immunohistochemistry (magnification, 40x). **(B)** Double immunohistochemisrty staining for CD4^+^ (in brown) and CD8^+^ (in red) T cells (magnification, 10x). **(C)** Immunohistochemistry staining for PD-1 highlights numerous T-follicular helper cells within the germinal centers (stronger expression) as well as in the interfollicular areas (magnification, 10X). **(D–F)** Immunohistochemical stains for CD20^+^ **(D)**, IgM^+^ **(E)**, and IgG^+^ cells **(F)** showing a marked increase of IgM positive plasma cells over IgG (magnification, 4x).

Finally, during admission at the NIH, a PET-scan was performed on the mother, confirming hypermetabolic generalized lymphadenopathy. In order to better define the nature of the lymphoproliferative process, an excisional biopsy of a left cervical lymph node was performed, which revealed an abundance of PD-1^+^ T follicular helper (T_FH_) cells, a striking predominance of IgM^+^ plasma cells (with few IgG^+^ plasma cells), and abnormal germinal centers (Figure 2). The proportion of CD4^+^ CXCR5^+^ CD45RA^−^ CD25^−^ T_FH_ cells was also markedly increased in circulation in the patient as compared to two healthy controls (41.7 vs. 13.7% and 16.6%).

Upon functional and genetic confirmation of APDS2, we initiated treatment with sirolimus in the mother to inhibit mTOR activation, and added IgG replacement therapy and *Toxoplasma* suppressive therapy with TMP-SMX. This treatment has resulted in improvement of the lymphadenopathy. She remains negative for CMV and EBV viremia by quantitative PCR. Her daughter has been started on TMP-SMX to prevent reactivation of *T. gondii*. At the time of her last follow-up visit (3 years and 3 months of age), she presented with typical immunological hallmarks of the disease, including an increased proportion of central memory CD4^+^ cells (44.1% of total CD4^+^ cells) and of T_EMRA_ CD8^+^ cells (36.1%), along with an elevated proportion of transitional B cells (31.6% of total CD19^+^ cells).

## Discussion

### Immune Deficiencies Reveal Critical Mechanisms Involved in Protection Against *T. Gondii* Infection

*T. gondii* is an obligate intracellular parasite that establishes a relatively benign, life-long infection with only immunocompromised hosts showing signs of clinical disease (12). Encephalitis and ocular infections are reported in secondary immunodeficiencies due to HIV, chemotherapy and post solid organ or hematopoietic stem cell transplant. Humans and mice act as intermediate hosts and are infected either by ingesting undercooked meat laden with *T. gondii* tissue cysts or by drinking oocyst contaminated water. Tachyzoites released from oocysts or tissue cysts then replicate in intestinal epithelium and myeloid cells can disseminate the infection (13).

Murine models suggest that different lymphocyte subsets are involved in the acute and chronic phases of parasite control. The acute phase of infection is mediated principally by macrophages, dendritic cells, NK and CD4^+^ cells (14–16). Upon engagement of the chemokine receptor 5 (CCR5), Toll-like receptors (TLR) 2 and 11, and the inflammasome sensors NLRP1 and NLRP3 by *Toxoplasma* molecules, antigen presenting cells are activated to produce IL-12 and IL-18 (17–19), triggering IFN-γ production by CD4^+^ and NK cells. In turn, IFN-γ upregulates production of reactive oxygen species (ROS) in macrophages thereby inhibiting parasite replication (20, 21). On the other hand, the memory immune response during chronic infection or upon antigenic challenge is driven by IFN-γ producing CD8^+^ T cells (22).

Identifying human primary immunodeficiencies that are associated with *Toxoplasma* infection is critical for developing an understanding of host defenses against this parasite. A review of the literature yields 24 cases with a diverse set of primary immunodeficiencies that predispose human hosts to severe toxoplasmosis (Table 2). The heterogeneity of these diseases suggests that multiple immune system defects may contribute to toxoplasmosis pathogenesis.

**Table 2:** Cases of toxoplasmosis in patients with primary immune deficiencies.

**Patient #**	**PID/Age at diagnosis/mutation**	**Age at infection/site of *Toxoplasma* infection**	**Diagnostic method of *Toxoplasma* detection**	**Clinical signs/co-morbidities**	**Treatment**	**Outcome**	**References**
1	APDS1/NR/NR	9 m/NR	NR	NR	NR	NR	(8)
2	APDS2/NR/NR	36 y/ocular	NR	NR	NR	NR	(9)
3	Hyper IgM/2 y/none (absent CD40L on flow)	10 y/cerebral	*Toxoplasma* tachyzoites seen on brain biopsy	Fever, headache, Babinski, anorexia, papilledema, facial pain	Pyrimethamine, sulfadiazine, dexamethasone	Alive	(23)
4	CD40L deficiency/41 y/p.R11X	41 y/cerebral	*Toxoplasma* tachyzoites and occasional cysts on brain biopsy	Recurrent impetigo, Gait disturbance, left sided weakness, sensory disturbance, hemiparesis, sensory loss	Sulfadiazine, pyrimethamine, clarithromycin, clindamycin	Alive	(24)
5	CD40L deficiency/1.4 y/p.C218X	2.8 y/cerebral	Positive *Toxoplasma* PCR on CSF	Diarrhea, lethargy, fever, gait disturbance	Sulfadiazine, azithromycin, G-CSF	Deceased	(25)
6	CD40L deficiency/5 m/p.V237E	9 y/cerebral	*Toxoplasma* tachyzoites on post mortem brain analysis	Nausea, vomiting, seizures, reduced reflexes, dyskinesia	Acyclovir, IFN- α (treated for possible viral encephalitis)	Deceased	(26)
7	CD40L deficiency/NR/NR	NR/cerebral	NR	NR	NR	NR	(27)
8	NFKB2 deficiency/2 y/p.R853X	6 y/cerebral and retina	Positive *Toxoplasma* PCR on CSF	Bilateral visual impairment	Sulfadiazine, pyrimethamine, systemic and ocular steroids	Alive	(28)
9	IL-12Rβ1 deficiency/37 y/c.[1745_1746delinsCA]+[1483+182_1619-1073del]	NR/retina	NR	NR	NR	Alive	(29)
10	IL-12Rβ1 deficiency/29 y/p.L77p	NR/choroid and retina	NR	NR	NR	Alive	(29)
11	IFN-γR1 deficiency/29 y/p.I87T (homozygous)	NR/cerebral	NR	NR	NR	Alive	(30)
12	Anti- IFN-γ auto-antibody/65 y/NR	63 y/cerebral	*Toxoplasma* tbradycoites on brain biopsy	None reported	Sulfadiazine, pyrimethamine	Alive	(31)
13	STAT1 GOF/20 y/c.1154C>T	2 y/retina (thought to be congential)	Chorioretinal scar in left eye (thought to be pathognmonic for *Toxoplasma*)	NR	NR	Alive	(32)
14	TAP1 deficiency/14 y/c.C522T	14 y/ocular	Positive *Toxoplasma* serology (IgM)	Monoliteral reduction in acuity, cheratitis, anterior uveitis, chorioretinitis, retinal detachment, ultimate loss of vision	Sulfadiazine, pyrimethamine	Alive	(33)
15	TAP1 deficiency/6 y/c.C1312T	14 y/pneumonitis	Positive *Toxoplasma* serology (IgM)	Granulomatous facial skin lesions, fevers, respiratory distress	Pyrimethamine, sulphadoxin	Alive	(34)
16	CVID/46 y/NR	46 y/ocular then disseminated, 50 y/hepatomegaly, 52 y/LAD	46 y-positve Sabian-Feldman dye test; 50 y—positive IFA; 52 y—*Toxoplasma* seen on lymph node biopsy	Uveitis/decreased mitogen T cell responses	Sulfadiazine, pyrimethamine, leucovorin	Alive	(35)
17	CVID/NR/NR	38 y/cerebral	*Toxoplasma* cysts on brain biopsy	Diplopia, hemianopia, hemiparesis, aphasia/PML	NR	NR	(36)
18	CVID/“adulthood”/NR	52 y/cerebral	Positive IHC, pseudocysts on brain biopsy	Spastic tetraparesis, dysarthria, dysphagia/autoimmune hemolytic (received steroids 4 weeks prior to diagnosis) and LGL	Sulfadiazine, pyrimethamine	Alive	(37)
19	CVID/19 y/NR	40 y/cerebral	Positive IHC on brain biopsy	Hemiparesis, loss of nasolabial fold, dysphasia/chronic steroids for necrotizing autoimmune enteritis	Sulfadiazine, pyrimethamine	Alive	(38)
20	Good syndrome/43y (thymoma)/NR	54 y/cerebral and then disseminated	Positive *Toxoplasma* PCR on brain biopsy	Headache, visual disturbance, right sided facial weakness	Sulfadiazine and pyrimethamine first, then atovaquone added	Alive	(39)
21	Mother with Good syndrome (diagnosed 5 years after delivery)/NR/NR	5 m	Positive *Toxoplasma* serology (IgM and IgG)	Nystagmus			(40)
22	Nijmegen breakage syndrome/NR/NR	15 y/disseminated	Tachyzoites on BAL, positive *Toxoplasma* PCR on blood and BAL	Acute chest pain/HSCT	Intravenous trimethoprim-sulfamethoxazole, clindamycin	Deceased (39 days post transplant)	(41)
23	Omenn syndrome/2 m/NR	2 m/NR	Positive *Toxoplasma* serology on blood and CSF (IgM)	NR	HSCT	NR	(42)
24	ORAI1/NR/NR	8 m/cerebral	NR	NR/CMV infection	NR	Deceased	(43)

*NR, not reported*.

### Defects of IL-12/IFN-γ Loop

The IL-12/IFN-γ loop facilitates T cell activation and proliferation and intracellular macrophage-mediated parasite killing. Upon T cell receptor (TCR) recognition of a MHC:*Toxoplasma* peptide complex, T lymphocytes are activated and express the CD40 ligand (CD40L) molecule on the cell surface, enabling T cell interaction with macrophages and dendritic cells that constitutively express CD40. CD40L:CD40 signaling activates the non-canonical NF-κB pathway (NFKB2), which increases the cell surface expression of the co-stimulatory molecules CD80/86 (44). In addition, NFKB2 upregulates IL-12 production in activated macrophages (45). The ligation of the IL-12R on T lymphocytes by IL-12 leads to IFN-γ release. In turn, ligation of the IFN-γR on macrophages leads to activation of the JAK/STAT pathway, with phosphorylation, dimerization and nuclear translocation of Signal Transducer and Activator of Transcription 1 (STAT1), which helps produce anti-*Toxoplasma* effectors like intracellular ROS.

Review of the literature identified a total of 10 patients with severe *Toxoplasma* infection involving five distinct gene defects along the IL-12/ IFN-γ loop: four cases of CD40L deficiency, one case of heterozygous mutation in *NFKB2*, two cases of IL-12Rβ1 deficiency, one case of STAT1 gain-of-function mutation, and two cases of autosomal recessive IFN-γR1 deficiency (23–30). Furthermore, anti-IFN-γ autoantibodies were demonstrated in one patient with severe toxoplasmosis (31). Interestingly, disruption of IFN-γ signaling (receptor deficiency or auto-antibody production) and CD40L deficiency result in more severe disease, as these defects were associated with *Toxoplasma* encephalitis.

There is evidence that TNF-α may also provide protection against *T. gondii*. In particular, early work by Jack Remington established that regulation of TNF expression determines susceptibility to toxoplasmic encephalitis in mice (46). Furthermore, Jannsen et al. demonstrated in patients with partial IFN-γR1 deficiency that the addition of TNF-α can limit *T. gondii* replication *in vitro* (47). The important role of TNF-α in parasite control was also confirmed by the observation that treatment with infliximab may lead to reactivation of cerebral toxoplasmosis (48).

As mentioned previously, CD8^+^ cells mediate the memory response by recognizing *Toxoplasma* antigens in the context of MHC class I molecules. Transporter associated with antigen processing (TAP) is critical in transporting peptides to the lumen of the endoplasmic reticulum and facilitating peptide loading onto MHC class I molecules. Ocular and pulmonary toxoplasmosis has been reported in two patients with TAP1 deficiency (33, 34). On the other hand, *Tap1*^−/−^ mice succumb to infection faster than *Cd8*^−/−^ mice, and CD4^+^ T cells and NK lymphocytes from *Tap1*^−/−^ mice produce reduced amounts of IFN-γ (49), indicating that TAP deficiency affects both acute and chronic phases of *Toxoplasma* infection.

### Common Variable Immunodeficiency

Humoral responses are not thought to play an essential role in limiting *Toxoplasma* replication. Three cerebral and one case of disseminated toxoplasmosis in common variable immunodeficiency are published (35–38). However, the clinical history and laboratory findings of these cases, characterized by chronic steroid administration for necrotizing autoimmune enteritis, development of large granular leukemia, progressive multifocal leukoencephalopathy and significant reduction in mitogen-induced T cell proliferation, are suggestive of co-existing cellular defects.

### Good Syndrome

Patients with Good Syndrome have thymomas with combined cellular and humoral immunodeficiency and autoimmunity. Cerebral and disseminated *Toxoplasma* infection was reported in one patient with Good syndrome (39), and *Toxoplasma* reactivation in pregnancy resulting in congenital toxoplasmosis has been described in another patient with the same disease (40). Further studies are needed to characterize the molecular and cellular mechanisms predisposing to *Toxoplasma* infection in this patient population.

### Other T Cell Immunodeficiencies

Fulminant and fatal reactivation of *T. gondii* infection has been reported early after allogeneic hematopoietic cell transplantation (HCT) in a patient with Nijmegen breakage syndrome (41). In addition to the underlying immunodeficiency, both the *Toxoplasma* seronegative status of the donor, and use of immunosuppressive therapy pre- and post-transplantation may have contributed to the fatal outcome in this patient. Invasive toxoplasmosis after allogeneic HCT has bene reported in 4–6% of seropositive recipients, with an estimated mortality rate as high as 60–90% (50, 51).

In spite of the severity of T cell immunodeficiency, few cases of toxoplasmosis have bene reported in patients with severe combined immune deficiency (SCID) and related disorders. The rarity of this association is probably related to the fact that infants with SCID die early in life unless immune reconstitution is achieved with HCT. However, cerebral toxoplasmosis has been reported in an infant with Omenn syndrome (42) and in another infant with severe T cell immunodeficiency due to calcium flux defect (43).

### APDS

**Figure 3:**
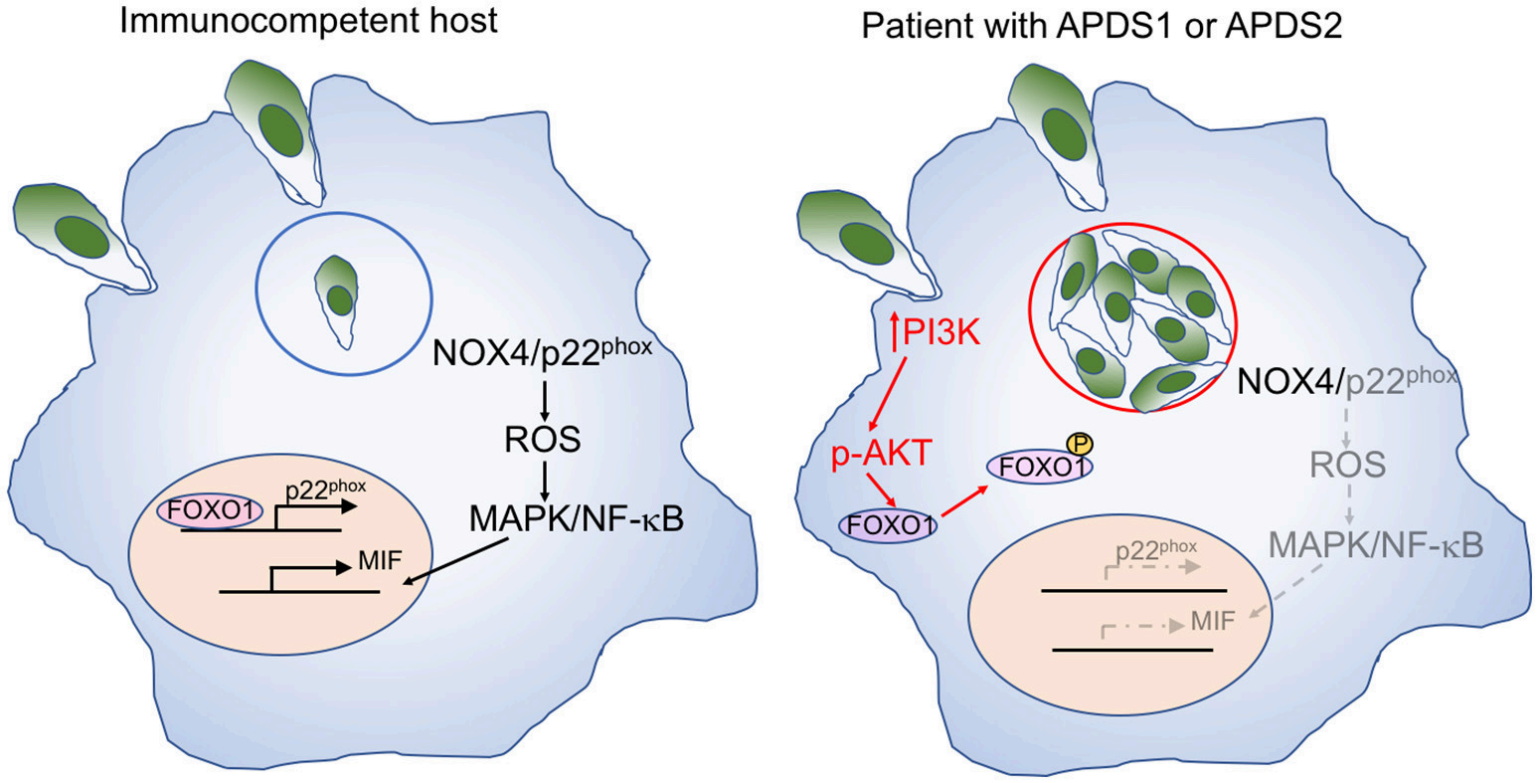
Mechanisms of macrophage-mediated response against *Toxoplasma*, and effects of increase PI3K signaling. **Left:** In immunocompetent hosts, intracellular *Toxoplasma* infection with tachyzoites within parasitophorous vesicles (blue circle) elicits a macrophage response mediated by NOX4 and p22^phox^. Expression of the latter is controlled by the FOXO1 transcription factor. Activation of the NOX4/p22^phox^ complex allows generation of reactive oxygen species (ROS), activation of MAP kinase (MAPK), and NF-κB signaling, and production of the pro-inflammatory macrophage inhibitory factor (MIF). **Right:** In patients with APDS1/2, increased PI3K signaling induces AKT phosphorylation, which in turn mediates phosphorylation of FOXO1, impairing p22^phox^ gene expression (in gray). This causes reduced production of ROS, defective activation of MAPK and NF-κB, and impaired production of MIF (all in gray) in response to *Toxoplasma* infection. Furthermore, the favorable metabolic environment supported by enhanced PI3K activity promotes intracellular replication of *Toxoplasma* tachyzoites.

*Toxoplasma* infection has been previously reported in a 9-month-old infant with APDS1 (8), and ocular involvement has been described in a 36-year-old patient with APDS2 (9). *T. gondii* may evade host defense by inducing the activation of the PI3K/AKT signaling pathway which reduces intracellular reactive oxygen species (ROS) through NOX4 suppression (52) (Figure 3). Active AKT phosphorylates and thereby inactivates the transcription factor FOXO1, thereby preventing p22^phox^ transcription. The resultant decrease of ROS in macrophages leads to reduced activation of AP-1, MAPK, and NF-κB and decreased production of macrophage migration inhibitory factor (MIF), a pro-inflammatory cytokine that plays an important role in the immune response to *Toxoplasma* (53).

Moreover, AKT phosphorylation promotes glycolysis and activation of the amino acid sensor mechanistic target of rapamycin complex 1 (mTORC1), thereby creating an intracellular metabolic environment that is favorable to the intracellular proliferation of *Toxoplasma* parasites, that can utilize micronutrients for their own replication cycle.

## Concluding Remarks

We have described two patients, a mother and daughter, with an essential donor splice site mutation of *PIK3R1* and disseminated and congenital toxoplasmosis, respectively. Few cases of parasitic infections have been previously reported in patients with APDS, including diarrhea due to *Cryptosporidium parvum* or to *Giardia lamblia* (8–10). This is the first time that systemic and severe congenital toxoplasmosis are reported in a mother and child with APDS2, an immune defect that may favor *Toxoplasma* replication. For patients with APDS who are considered for HCT, both donor and recipient *Toxoplasma* serology must be included in the screening work-up. Finally, the identification of severe *Toxoplasma* infection in patients with inborn errors of immunity has helped identify cellular and molecular mechanisms that are critically involved in the immune response against this parasite.

## Ethics Statement

This study was carried out in accordance with the recommendations of the Declaration of Helsinki and consistent with Good Clinical Practice and the applicable regulatory requirements, according to protocol 05-I-0213 which was approved by the IRB of the National Institutes of Health and is registered at ClinicalTrials.Gov as NCT00128973. Written informed consent has been obtained from the patient for the publication of this case report.

## Author Contributions

DK, IM, ZA, LN, and MG wrote the manuscript. IM, AH, SD, OD, ZA, NR, and NH identified the patient's condition and provided clinical care. AD, JN, JS, SR, and GU performed experiments. SP performed tissue pathology studies. LN supervised and contributed to the whole work.

### Conflict of Interest Statement

The authors declare that the research was conducted in the absence of any commercial or financial relationships that could be construed as a potential conflict of interest.
